# The Influence of Feeding with Colostrum and Colostrum Replacer on Major Blood Biomarkers and Growth Performance in Dairy Calves

**DOI:** 10.3390/vetsci10020128

**Published:** 2023-02-07

**Authors:** Ramune Grigaleviciute, Rita Planciuniene, Ieva Prikockyte, Eivina Radzeviciute-Valciuke, Austeja Baleviciute, Augustinas Zelvys, Aukse Zinkeviciene, Vilma Zigmantaite, Audrius Kucinskas, Paulius Matusevicius, Povilas Kavaliauskas

**Affiliations:** 1 Biological Research Center, Lithuanian University of Health Sciences, Tilzes Str. 18/7, LT-47181 Kaunas, Lithuania; 2 Department of Animal Nutrition, Lithuanian University of Health Sciences, Tilzes Str. 18, LT-47181 Kaunas, Lithuania; 3 Institute of Microbiology and Virology, Lithuanian University of Health Sciences, Tilzes Str. 18, LT-47181 Kaunas, Lithuania; 4 Kaunas Hospital, Lithuanian University of Health Sciences, Josvainiu Strg. 2, LT-47144 Kaunas, Lithuania; 5 Department of Immunology, Centre for Innovative Medicine, Santariskiu Str. 5, LT-08410 Vilnius, Lithuania; 6 Toxicology Unit, Institute of Environmental Medicine, Karolinska Institutet, Solnavägen 1, 171 77 Solna, Sweden; 7 Joan and Sanford I. Weill Department of Medicine, Weill Cornell University, 1300 York Avenue, New York, NY 1109, USA; 8 Department of Microbiology and Immunology, University of Maryland Baltimore School of Medicine, Baltimore, MD 21201, USA; 9 Institute of Infectious Diseases and Pathogenic Microbiology, Birstono Str. 38A, LT-59116 Prienai, Lithuania

**Keywords:** bovine colostrum, calf, biomarkers, immunomodulation, neutrophils, maternal milk

## Abstract

**Simple Summary:**

Bovine colostrum (BC) is known to exert immunomodulatory activity by stimulating mucosal immune responses as well as providing the passive transfer of immunoglobulins. Despite the evident role of BC-derived immunoglobulins for protection of the calves, the multifaceted BC activity on neonate calves could not be fully explained by passively acquired immunoglobulins. Therefore, it is critical to better understand the role of other, non-immunoglobulin-derived bioactive constituents on the growth and development of neonate calves. Our data demonstrated that colostrum administration is important for the growth performance of dairy calves as well as changes in major blood biomarkers. The timing of colostrum administration results in a decremental effect on the body mass growth in calves.

**Abstract:**

Bovine colostrum (BC) is the first milk produced by lactating cows after parturition. BC is rich in various amino acids, proteins, and fats essential for the nutrition of the neonate calves. Despite the evident beneficial effect of BC on calves, the effect of BC on blood biomarkers is poorly understood. Calves that received BC showed significantly higher body mass at days 7 and 30 (38.54 kg and 43.42 kg, respectively) compared to the colostrum replacer group (*p* = 0.0064). BC induced greater quantities of blood neutrophils (0.27 × 10^9^/L) and monocytes (4.76 × 10^9^/L) in comparison to the colostrum replacer (0.08 and 0.06 × 10^9^/L, respectively) (*p* = 0.0001). Animals that received BC showed higher levels of total serum protein (59.16 g/L) and albumin (29.96 g/L) in comparison to the colostrum replacer group (44.34 g/L and 31.58 g/L, respectively). In addition, BC induced greater intestinal mucus production in the Wistar rat model. Collectively, these results demonstrate that BC is important for the growth of calves and that it provides a significant beneficial effect on morphological and biochemical blood parameters.

## 1. Introduction

Bovine colostrum (BC) is the first milk produced by lactating cows after parturition [1]. BC is rich in numerous components that ensure full nutrition, and enhance the growth, health and development of the calves [2,3,4,5]. Previous studies have identified that BC is predominantly enriched in immunoglobulins, lactoferrins, growth hormones, oligosaccharides, and numerous bioactive proteins and small molecules that are often secreted in a time-dependent manner [5,6,7]. Furthermore, various regulatory microRNA, interleukins, and even intact T and B lymphocytes are present in BC. These biologically active constituents are known to play a protective immunomodulatory role [8,9]. Historically, many efforts were made to understand the biochemical composition of BC as well as the effects of prolonged feeding, although the biological significance of early processes induced by the first hours of feeding with BC by neonate calves remains poorly understood [10].

BC is specifically enriched in fat, proteins and soluble carbohydrates that ensure the proper nutrition of neonate calves during first month postpartum. On the other hand, the exact nutritional requirements as well as the caloric intake provided by BC are not well defined. Several studies have reported the variable nutritional profiles of BC that are dependent on the BC collection time, geographic location and age of the animal [2,3,4,5]. Moreover, it is not always possible to cause complete BC deprivation in experimental animals to fully understand the biological role of BC; therefore, the careful selection of BC replacer controls is critical for the nutrition studies. Therefore, it is crucial to use a well-defined small vertebrate experimental animal model, such as Wistar rats, to better understand the nutritional potency of BC [11,12,13].

Various methods were employed in understanding the minor proteinaceous and non-proteinaceous composition of BC. Previous studies have shown that BC is highly enriched in immunoglobulins, and it is historically thought to play a major role in the passive immunization of calves. The composition of BC is non-static, and it is believed that the secreted bioactive molecules are being detected in variable abundance depending on the colostrum collection time, suggesting that the biological effect conferred by BC could be highly affected by the length and duration of BC feeding. Several studies have also shown that timely administration of colostrum to neonate calves results in higher circulating blood IgG titers, thus suggesting that BC is the main factor driving the serum IgG concentration [9]. It is believed that the colostrum-derived IgG in the intestinal track can be translocated to the blood via the FcγR receptor-mediated pathway, conferring passive protection from pathogens [12,13,14]. Besides immunoglobulins, BC is rich in various antimicrobial peptides, defensins, and other proteins, although their role in innate or adaptive immune responses in calves has not been previously investigated.

The crosstalk between intestinal microbiota and host mucosal layers in neonate calves is paramount for the development of the calves and their further survival. BC, as the first food that is received by calves, shapes the development of intestinal microbiota by enhancing the probiotic bacterial populations and suppressing colonization with pathogens. Previous studies have demonstrated that cows can passively transfer probiotic bacteria to calves via feeding with colostrum [15,16,17,18,19]. Moreover, BC is highly enriched by various non-digestible oligosaccharides, antimicrobial peptides and various biomolecules that can exert anti-inflammatory activity, thus enhancing the colonization of the normal microbiota in calves [19,20,21]. The restriction of growth and colonization of pathogenic microorganisms in the intestinal tract provides immunostimulatory and anti-inflammatory signals, resulting in the homeostasis of innate and adaptive immune cells and their responses. Moreover, the immunomodulatory activity of BC was further confirmed by using various laboratory models and human clinical trials focused on various inflammatory diseases [22,23].

Historically, numerous efforts were made to understand the biological effect, molecular mechanisms as well as cellular targets that are activated in response to BC feeding [1]. Many studies were focused on understanding the effects of prolonged BC feeding on the health of the calves, although the effects of BC received for the first six hours were never compared with commercial colostrum replacers that are widely used in the veterinary and agricultural fields.

In this study, we aimed to systematically compare the changes of major blood biomarkers induced by feeding with colostrum and colostrum replacer administered for the first six hours postpartum in calves and compare the findings using a well-described Wistar rat nutrition model.

## 2. Materials and Methods

### 2.1. Study Animals and Husbandry Practices

Studies involving animals were carried out in accordance with the recommendations of the Guide for the Care and Use of Laboratory Animals of the National Institutes of Health. The study protocols were approved by the State Food and Veterinary Service (SFVS) animal care and welfare committee (approval number: No G2-164). Holstein Friesian breed male (*n* = 4) and female (*n* = 6) calves were kept in the individual stall with at least 1.5 m^2^ of floor space for each animal, and artificial lighting is kept from 8 a.m. to 5 p.m. Manure, slurry and uneaten feed are regularly removed. All animals were inspected at least twice a day. Calves were fed according to their age, weight, behavior, and physiological needs. All calves were weighted at least once a week if it was needed, and more often.

Six-week-old female and male Wistar rats (*n* = 42) were obtained from the Lithuanian University of Health Sciences animal breeding facility and were housed under standard conditions. Rats were kept according to animal welfare recommendations, 12 h day and night cycle, room temperature of 21 °C and 50% humidity. Water and feeding, commercial feed for experimental animals *ad libitum.*

### 2.2. Treatment Groups

During pregnancy, Holstein Friesian cows (*n* = 10), two–four years of age, (weight 650–770 kg., 2–3 parity of the dam), were randomly selected from the farm located in Lithuania (Lytagra Agriculture company, Naujieji Bernatoniai, Kaunas District, Lithuania). After parturition, neonate calves were immediately separated from the cow and randomly divided into two groups: the CF, colostrum fed, group received a total amount of colostrum (3.2–3.8 L/calf) administered every 3 h (*n* = 5) divided into two times (total 6 h); and the CNT (control) group (*n* = 5), which received commercial colostrum replacer at the same rate as that of the CF group. Before feeding, colostrum was pre-warmed to 38 °C using a water bath and administered to the calves through the esophageal tube. The colostrum replacer was a commercial product (Euronovo, La Perrière, France). The feeding was continued for 6 h, and animals were returned to individual stalls. Further calves were fed according to the regimen and feeding program established on the farm (whole milk, followed by solid chew after one week postpartum). The powder of colostrum replacer was mixed with warm water (about 38 °C) until completely dissolved, and then given to the calf through an esophageal tube. The experimental animals were observed on days 1, 7 and 30, and their weight was measured, and venous blood was also collected and used for analysis.

#### 2.2.1. Blood Sampling and Analysis

The calves blood samples were collected from the jugular vein by performing venipuncture using potassium EDTA and clot activator-treated tubes (BD Vacutainer, BD, New York, NY, USA). When serum was needed, blood samples were centrifuged for 2500× *g*, for 20 min in a refrigerated centrifuge (4 °C). Serum was separated and stored at −80 °C until the day of the experiments. Total protein (TP), albumin (ALB), high-density lipoproteins (HDL), urea, creatinine, bilirubin (BIL), aspartate aminotransferase (AST), alanine aminotransferase (ALT) and alkaline phosphatase (ALP) were measured from serum samples using the SELECTRA Junior (Elitech, Spankeren, The Netherlands) biochemical analyzer. EDTA-treated blood was used for the determination of total white blood cells (WBC), lymphocytes (LYM), monocytes (MON), neutrophils (NEU) and eosinophils (EOS) by using the VetScan HM (Abaxis, Kalamazoo, MI, USA) morphological analyzer.

#### 2.2.2. Colostrum Collection

Colostrum samples were collected from healthy Holstein Friesian breed cows (*n* = 10) raised on the farm (Lytagra Agriculture Company, Naujieji Bernatoniai, Kaunas District, Kaunas, Lithuania). Cows were randomly selected from first to third gestation to have similar colostrum quality. Colostrum fractions were collected every hour for four hours (namely, T1–4 h) postpartum. The colostrum samples collected from individual animals were then pooled together and homogenized by mixing for 5 min at 4 °C. Homogenized pooled colostrum fractions were then aliquoted into sterile tubes (5 mL each) and kept at −80 °C until the day of the experiments.

#### 2.2.3. Characterization of Nutritional Profiles of Colostrum, Colostrum Replacer and Milk

The colostrum, colostrum-replacer and milk samples were mixed with commercial stabilization reagent (Broad Spectrum Microtabs, Bentley Instruments, Chaska, MN, USA). The total concentration of fat, protein and lactose was measured using milk analyzer (LactoScope 300, Perkin Elmer, Waltham, MA, USA).

### 2.3. Wistar Rat Model

Before the experiments, rats were randomly divided into seven groups, each containing six animals in total (three animals per experimental group, two independent experiments). The study. The animals received separate fractions of colostrum (10 mL/kg, two times per day), collected at different time points (named T1–T4 h, four groups in total) post parturition, commercial colostrum replacer (10 mL/kg, two times per day), milk (10 mL/kg, two times per day) and saline (10 mL/kg, two times per day) by oral gavage for 7 days. During the period of the experiments, Wistar rats received standard laboratory rodent food (Altronim, Lage, Germany) and water *ad libidum* [11,12].

For blood composition or body weight analysis in response to the colostrum or control treatments, Wistar rats received pooled colostrum (pooled fractions collected 1–4 h). Body weight and survival were monitored daily as outcome variables and animals were euthanized on day 7. The intestine and blood samples were collected for further analysis.

### 2.4. Tissue Collection and Histological Analysis

The Wistar rat intestine samples were processed as described by Naour et al. [24]. Briefly, the experimental animals were euthanized, and intestinal tissue was dissected and placed in ice-cold phosphate buffered saline (PBS). Intestine samples were rapidly rinsed in PBS and flushed using ice-cold modified Bouin fixative (5% acetic acid, 50% ethanol in deionized water). The Swiss-Roll technique was then applied, and samples were placed in tissue processing cassettes and fixed overnight in 10% (*v*/*v*) neutral formaldehyde (Sigma-Aldrich, St. Louis, MO, USA).

Tissue samples were then processed in an automatic tissue processor, where paraffin was embedded and sectioned. Tissue sections were then stained with hematoxylin-Eosin stain to characterize tissue architecture and Alcian blue staining to visualize mucins. Tissue sections were then evaluated by a pathologist blinded by the experimental design.

The thickness of the mucus layer was measured using ImageJ software (NIH, Bethesda, MD, USA). Briefly, the histological images were processed, and the microscopy size bar length was used for the normalization of the pixel count. Then, subsequently, the Alcian blue-stained mucus was measured in at least five different images, and the average length (mm) was then determined.

### 2.5. Statistical Analysis

Data were analyzed using GraphPad Prism (GraphPad Software, San Diego, CA, USA). The statistical significance was analyzed using one-way ANOVA and Mann–Whitney tests. Data were considered significant when *p* < 0.05.

## 3. Results

### 3.1. Body Mass Changes in Calves

**Figure 1:**
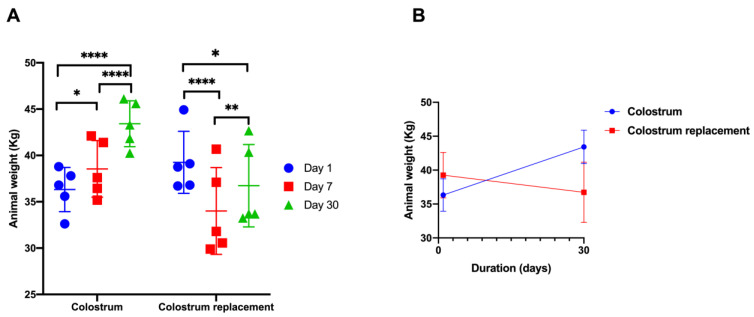
The effects of timely administration of colostrum result in increased body weight in the calves. The experimental animals received colostrum (*n* = 5) or artificial colostrum replacement (*n* = 5) for the first 6 h after birth. Panel (**A**) demonstrates the changes in body mass during on day 1, 7 and 30. Panel (**B**) demonstrates the body mass changes from day 1 to day 30. The changes in body weight were recorded at days 1, 7 and 30. The results are shown as mean ± SEM from five experimental animals. *^,^ **^,^ ****—denotes significance between time points of the same group.

To understand if timely administration of BC exerts an effect on neonate calves, we first evaluated the changes in body mass in calves undergoing the experimental feeding with BC or colostrum replacer (CR). To do so, we observed the body mass changes over the period of 30 days and compared them with calves that received control feeding. Interestingly, timely administration of BC results in increasing body mass acquisition over the period of 30 days. Strikingly, calves that received colostrum replacer showed decreased body mass (34.00 kg) at day 7 in comparison to day 1 (39.25 kg) (Figure 1A,B). At the final day of the experiment (day 30), the BC treated animals showed significantly higher body mass (43.42 kg) in comparison to the colostrum replacer-treated animals (36.73 kg) (*p* = 0.0064) (Figure 1A,B).

**Figure 2:**
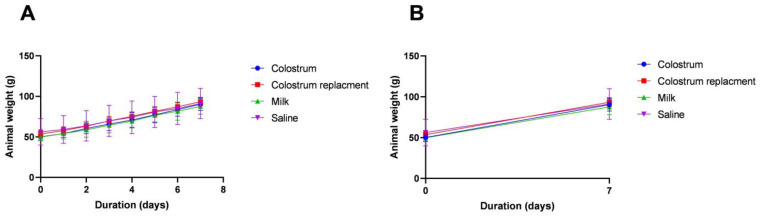
Feeding with colostrum has no significant effect on weight gain in Wistar rats (**A**,**B**). The 6-week-old Wistar rats received colostrum, an artificial colostrum replacer, milk and saline daily for 7 days. The weight changes were monitored. The figure shows mean ± SEM from six experimental animals.

Despite the fact that colostrum is significantly rich in fat (9.98%) and protein (13.63%), to our surprise, no significant changes in the growth of body mass were observed using the Wistar rat model treated with BC or colostrum replacer (Figure 2A,B and Figure S3). The BC did not induce significant changes in the body weights of rats over the course of 30 days.

### 3.2. Administration of Bovine Colostrum Modulates Lymphoid and Myeloid Blood Cell Populations in Calves

**Figure 3:**
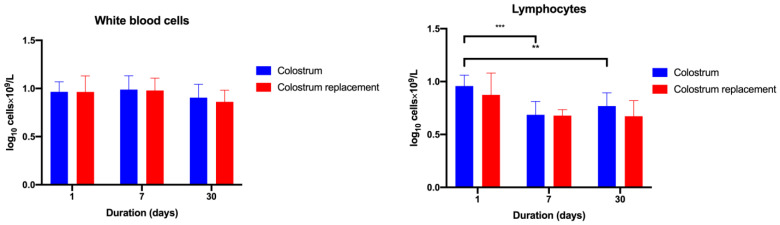
The administration of colostrum induces changes in lymphoid blood cell populations in calves. The experimental animals (*n* = 5 in each group) received colostrum or commercial colostrum replacement for the first 6 h after birth. On days 1, 7 and 30, the venous blood was collected and analyzed. The results are shown as mean ± SEM from five experimental animals. ** *p* < 0.05, *** *p* < 0.001.

Bovine colostrum or colostrum replacer failed to significantly affect the total counts of white blood cells over the period of 30 days (Figure 3). After this observation, we further questioned whether the BC or colostrum replacer administration could influence the changes in the numbers of lymphocytes. The total numbers of blood circulating lymphocytes significantly decreased at days 7 (5.02 × 10^9^/L) and 30 (6.05 × 10^9^/L) in BC-treated animals in comparison to day 1 (9.27 × 10^9^/L) *(p* = 0.0003). Furthermore, the administration of colostrum replacer did not result in significant changes in total lymphocyte counts (Figure 3).

**Figure 4:**
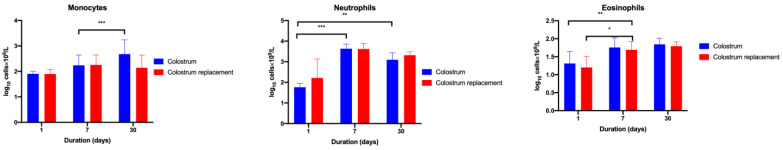
Administration of colostrum, no significant changes were observed in the calves’ blood monocyte, neutrophil and basophil populations. The experimental animals (*n* = 5 in each group) received colostrum or an artificial colostrum replacer for the first 6 h after birth. On days 1, 7 and 30, the venous blood was collected and analyzed. The results are shown as mean ± SEM from five experimental animals. * *p* < 0.05, ** *p* < 0.01, *** *p* < 0.001.

After characterizing the BC-induced changes in blood lymphocyte counts, we further investigated the myeloid cell populations. To do so, we characterized the numbers of monocytes and neutrophils in BC-treated animals and compared them with the colostrum replacer group. BC administration resulted in increasing numbers of monocytes and neutrophils at days 7 (0.27 and 4.76 × 10^9^/L) and 30 (0.70 and 1.52 × 10^9^/L) in comparison to day 1 (0.08 and 0.06 × 10^9^/L) (*p* < 0.0001). No significant changes in the populations of monocytes, neutrophils and eosinophils were observed between the colostrum and colostrum replacer groups (1.9–4 × 10^9^/L) (Figure 4).

The colostrum or colostrum replacer feeding failed to induce any significant changes in the blood cell populations in rats, suggesting that the feeding-induced changes observed in calves might be species-restricted (Figures S1 and S2).

### 3.3. The Effect of Early Colostrum Administration on Biochemical Renal Markers in Blood

**Figure 5:**
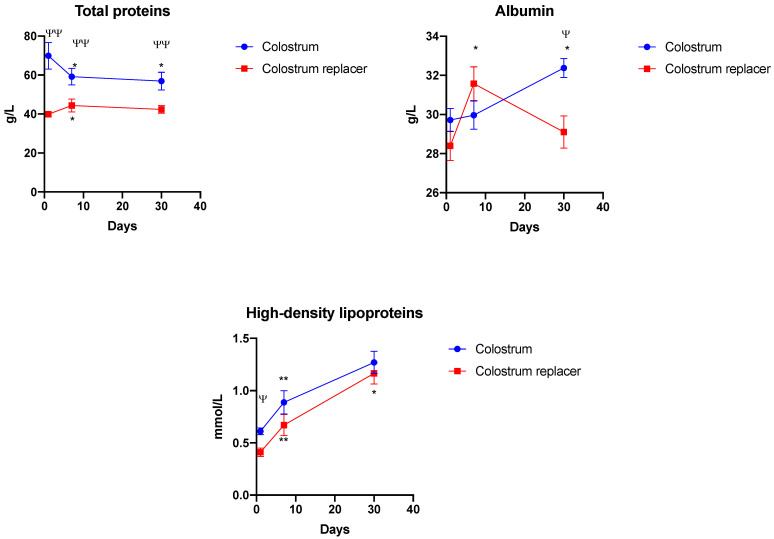
The changes in serum proteins and their composition in calves that received bovine colostrum or colostrum replacer. The early administration of colostrum results in a higher concentration of serum proteins in calves. The experimental animals (*n* = 5 in each group) received colostrum or an artificial colostrum replacer for the first 6 h after birth. On days 1, 7 and 30, the venous blood was collected and analyzed. The high-density lipoprotein concentration was quantified by measuring the cholesterol concentration. The results are shown as mean ± SEM from five experimental animals. Ψ – *p* < 0.05, ΨΨ – *p* < 0.01 indicates significance between experimental groups. * *p* < 0.05, ** *p* < 0.01 indicates significant changes between time points of the same group.

We then aimed to characterize the changes in total serum protein profiles among BC and colostrum replacer-treated animals (Figure 5). BC-treated animals showed significantly higher total serum proteins compared to the colostrum replacer group (*p* = 0.0001). The total serum protein in BC-treated animals was highest at day 1 (69.6 g/L) and significantly (*p* = 0.0113) decreased at days 7 and 30 (59.16 and 56.8 g/L, respectively) in comparison to day 1. Colostrum replacer-treated animals showed significantly lower total serum protein concentration at day 1 (39.8 g/L) in comparison to the BC group (*p* = 0.0427) (Figure 5). The total serum protein concentration rose significantly in the colostrum replacer group at day 7 (44.3 g/L) compared to day 1 (*p* = 0.0012). Finally, no significant differences were observed in total serum protein quantity in colostrum replacer-treated animals at days 7 and 30 (Figure 5).

After characterizing the changes in total serum proteins in BC and replacer-treated animals, we further evaluated the changes in serum albumin and high-density lipoprotein concentrations in both experimental groups (Figure 5). The serum albumin concentration steadily increased in the colostrum group over the whole duration of the experiment. Serum albumin concentration in BC-treated animals peaked at day 30 (32.3 g/L) and was significantly higher than the colostrum replacer group (29.1 g/L) (*p* = 0.0012). Interestingly, serum albumin concentration in the colostrum replacer group significantly peaked at day 7 (31.5 g/L) in comparison to day 1 (28.4g/L) (*p* = 0.0044) and then gradually decreased at day 30 (29.1 g/L). High-density lipoproteins in serum gradually increased in both experimental groups over the period of 30 days. BC-treated animals demonstrated significantly higher high-density lipoprotein concentration at day 1 (0.61 mmol/L) in comparison to the replacer group (0.41 mmol/L) (*p* < 0.0001).

**Figure 6:**
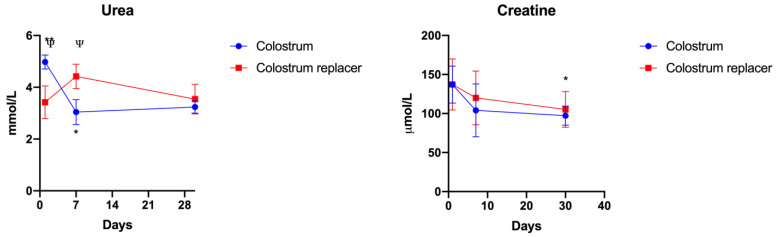
The changes in serum urea and creatinine concentration in calves that received a colostrum or colostrum replacer diet for the first 6 hours postpartum. The experimental animals (*n* = 5 in each group) received colostrum or an artificial colostrum replacer for the first 6 h after birth. On days 1, 7 and 30, the venous blood was collected and analyzed. The results are shown as mean ± SEM from five experimental animals. Ψ – *p* < 0.05 indicates significance between experimental groups. * *p* < 0.05, ** *p* < 0.01 indicates significant changes between time points of the same group.

After showing that early administration of BC is associated with higher serum protein levels in calves. We further estimated whether timely BC administration can affect the renal function in calves. To do so, we measured the changes in serum urea and creatinine over a period of 30 days in BC and colostrum replacer-treated animals (Figure 6). BC-treated animals showed significantly higher serum urea concentration at day 1 (4.98 mmol/L) in comparison to the colostrum replacer group (3.42 mmol/L) (*p* = 0.0039).

Interestingly, at day 7, the serum urea concentration in BC-treated animals significantly decreased and were lower (3.04 mmol/L) than the colostrum replacer group (4.42 mmol/L). No significant changes in serum urea concentration were observed at day 30. In addition, no significant changes were observed in serum creatine levels between colostrum or replacer-treated animals (Figure 6).

### 3.4. The Effect of Early Colostrum Administration on Biochemical Liver Markers in Blood

**Figure 7:**
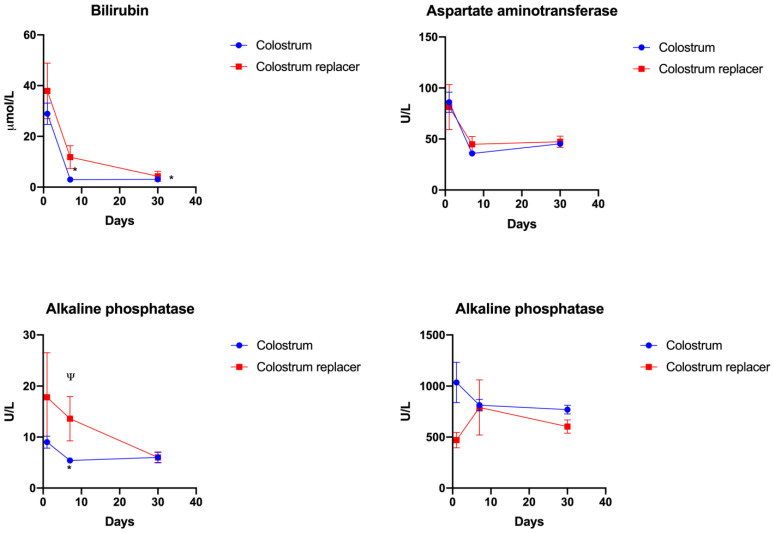
The effect of administration of colostrum or colostrum replacer on major liver markers in calves. The experimental animals (*n* = 5 of each group) received colostrum or artificial colostrum replacement for the first 6 h after birth. On days 1, 7 and 30, the venous blood was collected and analyzed. The results are shown as mean ± SEM from five experimental animals. *—denotes significant changes between time points of the same group (*p* < 0.05). Ψ – *p* < 0.05 indicates significance between experimental groups.

To elucidate the effect of colostrum administration in calves, we further characterized the changes in biochemical liver function markers (AST, ALT, ALP and bilirubin) in calves with BC or colostrum replacer administration (Figure 7).

The concentration of serum bilirubin similarly decreased in both experimental groups. The administration of colostrum resulted in significantly lower bilirubin concentrations at day 7 (2.96 µmol/L) and day 30 (4.28 µmol/L) in comparison to day 1 (28.9 µmol/L) (*p* = 0.0005) (Figure 7). The BC-treated group demonstrated a significantly decreased alanine transferase activity at day 7 (5.4 U/L) compared to the colostrum replacer group (13.6 U/L). Both experimental groups demonstrated no significant differences in aspartate aminotransferase or alkaline phosphatase activity at day 30 of the experiment (Figure 7).

Collectively, these results demonstrate that administration of colostrum results in improved liver function due to significantly decreased serum bilirubin (28.92–4.28 µmol/L) and alanine transferase activity (9.0-6 U/L) observed only in the colostrum-treated group.

### 3.5. Bovine Colostrum Enhances the Mucus Production in the Intestine of Wistar Rats by Colostrum-Collection in a Time-Dependent Manner

By combining histological staining with the Hematoxylin-Eosin staining as well as mucus-specific Alcian blue, we characterized the changes in the rat intestine after feeding with different fractions of colostrum collected at the first four hours postpartum (T 1–4 h). Commercial colostrum replacer (CR) and milk (M) served as feeding controls. Normal saline (NS) was used as a non-proteinaceous and non-nutritious control.

**Figure 8:**
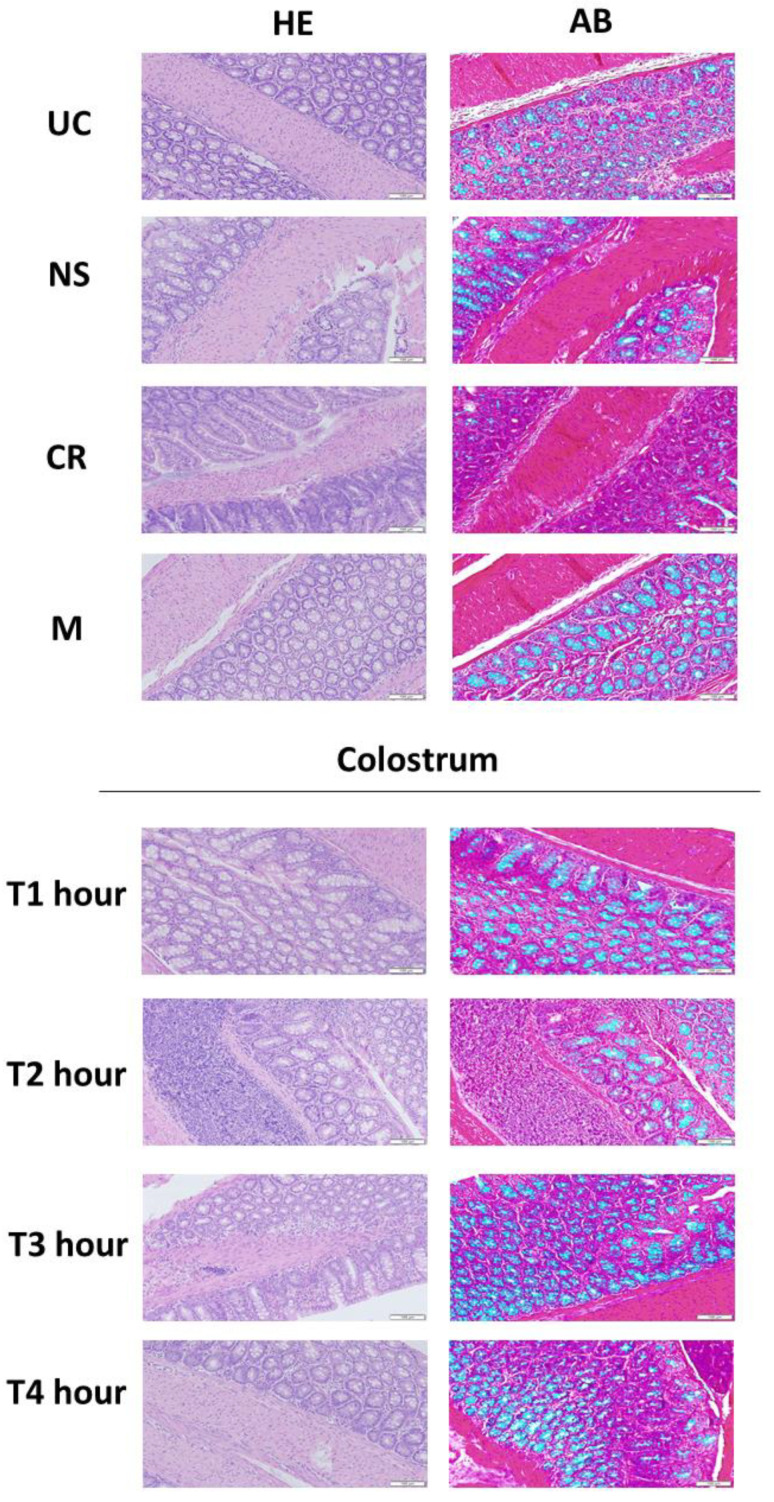
The feeding with bovine colostrum induced histological changes and mucus production in the intestine of Wistar rats in a colostrum collection fraction-dependent manner. Animals received colostrum collected at 1–4 h postpartum (T 1–4 h) as well as saline (NS), colostrum replacer (CR), and milk (M) that served as control. The feeding-induced changes were characterized by using hematoxylin-eosin (HE) and alcian blue (AB) staining. The size bar represents 100 µm.

**Figure 9:**
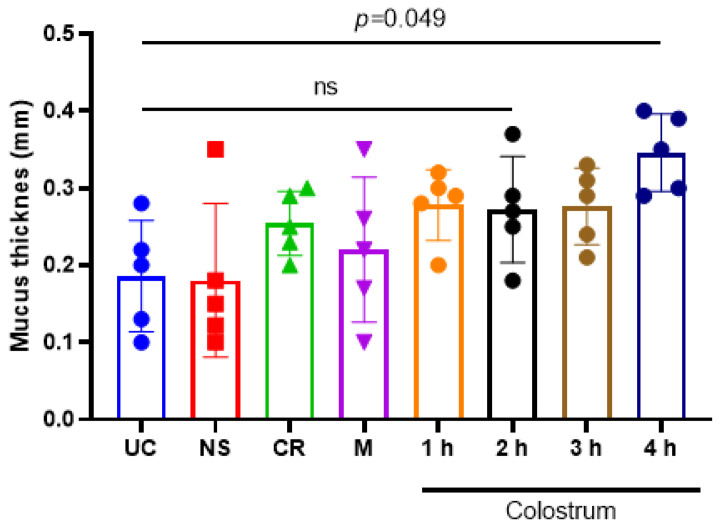
The bovine colostrum feeding resulted in a higher production of mucus in the Wistar rat intestine. The animals received experimental feeding with normal saline (NS), commercial colostrum replacer (CR), milk (M) and the diet-induced changes were compared with colostrum-treated animals or untreated control (UC). The thickness of intestinal mucus was determined after Alcian staining and ImageJ quantification. The results are shown as mean ± SEM from five experimental animals.

The BC administration resulted in changes in mucin production in the rat intestines (Figure 8). Colostrum feeding increased the thickness of the mucus layer by a fraction, depending on the manner. The animals that received colostrum fractions collected at 1–4 h postpartum showed a thicker mucin layer (0.25–0.3 mm) in comparison to the untreated control (UC) or milk treatment group (0.2–0.3 mm). Colostrum collected at 4 h postpartum induced significant mucus production, resulting in the highest (0.35 mm) mucus thickness in comparison to UC (*p* = 0.049) (Figure 9). The animals that received colostrum replacer (CR) had a visually smaller amount of goblet cells in comparison to a colostrum treatment group or UC. On the other hand, no significant changes were observed in intestinal villi morphology or tissue infiltrating immune cells among all treatment groups.

## 4. Discussion

Numerous studies have demonstrated the importance of bovine colostrum for the growth and development of neonate calves [8]. The complex bovine colostrum composition acts on various targeted systems in the organism and exerts powerful beneficial activity [25]. The studies of Puppel et al. showed that bovine colostrum is enriched in various subtypes of immunoglobulins, which play an important role in passive immunity [25,26]. Furthermore, colostrum is high in total protein concentration, including whey proteins, which serve as an important nutrient source for growing cattle [27].

Bovine colostrum (BC) is known to be rich in numerous nutrients that are important for the nutrition of the neonate calves. Godden et al. demonstrated that BC can contribute to body mass acquisition in neonate calves, demonstrating the high nutritional capacity of BC [8]. We demonstrated that pooled colostrum collected every hour for four hours is significantly richer in fat and protein in comparison to colostrum replacer or milk control. Interestingly, we demonstrated that colostrum has a significantly lower concentration of lactose than colostrum replacer or milk. Our study demonstrated that colostrum feeding resulted in steady weight acquisition in neonate calves, while delaying the administration of BC for the first 6 h postpartum resulted in significant weight loss profiles in the control group that received the commercial colostrum replacer. Moreover, BC contains numerous growth promoting factors, such as bovine epidermis growth factor (bEGF), insulin-like growth factor (bIGF) and others that are known to promote the growth of the animals [2,3,4,26]. Our data generated by using the Wistar rat nutrition model demonstrated that BC failed to induce the significant weight changes in the experimental animals, suggesting that the BC-mediated growth stimulatory activity observed in calves might not be fully dependent on the high nutritional profiles of BC colostrum. Similar results were observed by Nocek et al., where calves deprived of bovine colostrum showed altered body mass gain and decreased total serum protein concentration [28].

One of the major limitations of our study is our inability to fully recapitulate if the changes observed in body weight are truly not entirely dependent on the nutritional profiles of BC. Although animals received an experimental diet for only six hours postpartum and were returned to their standard diet right after this period, it is very unlikely that such a defined period of feeding would be able to induce the significant nutrition deficit in the colostrum replacer group in comparison to the BC group.

Bovine colostrum is known to exert immunomodulatory activity by stimulating mucosal immune responses and providing the passive transfer of immunoglobulins or other immunostimulatory and bioactive components. In addition to that, colostrum is rich in various cytokines, milk oligosaccharides and leukocytes that confer immunological protection as well as immunomodulatory properties [29,30,31]. After observing the beneficial effect of BC on the body mass in calves, we further postulated whether the early administration of BC exerts immunomodulatory activity on the myeloid and lymphoid cell populations in the blood. We successfully showed that the timely feeding of bovine colostrum is able to modulate neutrophil and monocyte populations in blood of the calves.

Immunoglobulins are one of the main constituents of total proteins in blood and are known to be involved in passive and adaptive immune responses in neonates. BC is highly enriched in various immunoglobulin-like proteins that could possibly be transferred to newborns via colostrum feeding. Furthermore, colostrum feeding is often associated with higher blood total protein quantities [32]. We observed that even feeding with colostrum results in a significantly higher total serum protein concentration compared to the colostrum replacer control group. Interestingly, we demonstrated that the serum albumin concentration in colostrum-treated calves increased gradually during the 30 days of the experiment, while the colostrum replacer group exhibited a spike in albumin at day 7 and then significant decrease at day 30. Similar results were also observed by Wilm et al. and Foster et al. [33,34,35]. The authors also correlated the total protein concentration in colostrum-treated animals with the serum IgG concentration as a marker of passive transfer in calves and were able to show that the animals treated with colostrum have a higher concentration of IgG in comparison to control animals.

The intestinal mucus layer plays an important role in the host’s nutrition, health and animal protection from pathogens [32]. The intestinal mucus layer provides a physical barrier separating the host microbiota from the surface of intestinal cells while still providing the ability to exchange nutrients, various small molecules, peptides and immunostimulatory components. Disruption of the intestinal mucus layer is often associated with various inflammatory diseases, malnutrition, or even severe infections, both in animals and humans [33]. Studies conducted in humans suffering from irritable bowel syndrome (IBS) demonstrated that bovine colostrum had a beneficial effect on restoring gut permeability and inflammation markers in patients that received colostrum [34]. Noel et al. demonstrated that human colostrum can enhance intestinal mucus production in a pediatric enteric model by upregulating the expression of occluding resultant proteins, in an important member of tight junction proteins [35]. In addition, Liu et al. showed that colostrum derived from goats can shape the intestinal microbiota and enhance the growth of *Lactobacillus*, which is known to be a powerful stimulus for *Muc2* gene expression and mucus production in the intestine [36]. The lack of well-established data on the calf intestinal permeability parameters as well as mucus layer variation remains the major challenge to characterizing the mucus stimulatory effect in calves. Therefore, we adapted a Wistar rat nutrition model with defined intestinal parameters. Our data demonstrate that colostrum administration in Wistar rats results in increased mucus production in the intestine in comparison to controls. The mucus-stimulatory effect exerted by colostrum depended on the colostrum collection time point, suggesting that mucus-stimulatory bioactive compounds are not present during all colostrum lactation periods.

## 5. Conclusions

This study successfully demonstrated that timely administration of colostrum in calves is important for the growth and development of neonate calves and morphological and biochemical blood markers. The lack of feeding with colostrum and the administration of colostrum replacer for the first six hours postpartum resulted in altered body weight parameters as well as morphological and biochemical blood parameters. Furthermore, the colostrum replacer alone cannot provide significant activity on the growth and biochemical blood markers in calves, demonstrating that colostrum feeding is critical for the health and growth of calves. Further studies are needed to understand better the molecular mechanisms of colostrum in these systems in calves.

## Data Availability

All data generated during this study is disclosed in the manuscript of supplementary files. The biological samples obtained during this study are available upon reasonable request from the corresponding author.

## Supplementary Materials

The following supporting information can be downloaded at: https://www.mdpi.com/article/10.3390/vetsci10020128/s1, Supplementary Figure S1 administration of colostrum fractions collected at different time points (1–4 h) after partition does not induce the significant changes in white blood counts in Wistar rats. Figure S2 administration of colostrum fractions collected at different time points (1–4 h) after partition does not induce the significant changes in granulocyte and monocyte counts in Wistar rats. Figure S3 the nutritional profiles of bovine colostrum, colostrum replacer and milk.
