# Supplementary material for: The Influence of Feeding with Colostrum and Colostrum Replacer on Major Blood Biomarkers and Growth Performance in Dairy Calves
Source: Vet Sci. 2023 Feb 7;10(2):128. doi: 10.3390/vetsci10020128 (PMC9965887; doi:10.3390/vetsci10020128)
Supplement: Supplementary file 1 [file vetsci-10-00128-s001.zip › vetsci-2195393-supplementary.pdf]

The Influence of the Feeding with Colostrum and Colostrum Replacer on Major Blood Biomarkers and Growth Performance in Dairy Calves

Ramune Grigaleviciute [1, 2], Rita Planciuniene [3], Ieva Prikockyte [4], Eivina Radzeviciute-Valciuke [5], Austeja Baleviciute [6], Augustinas Zelvys [5], Aukse Zinkeviciene [5], Vilma Zigmantaite [1], Audrius Kucinskas [1], Paulius Matusevicius [2] and Povilas Kavaliauskas [1,7,8,9]*.

Supplementary information

- Figure S1.
- Figure S2.
- Figure S3

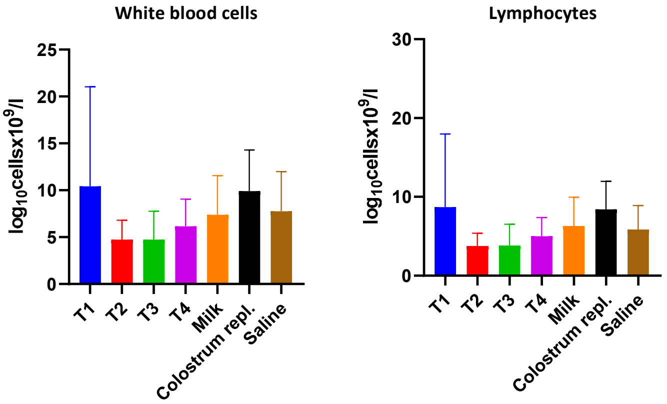

Figure S1. Early administration of colostrum fractions collected at different time points (1-4 hours) after partition does not induce the significant changes in white blood counts in Wistar rats. The 6 weeks old Wistar rats received colostrum, artificial colostrum replacer, milk and saline daily for 7 days. The weight changes were monitored. The results are shown as mean ±SEM from five experimental animals.

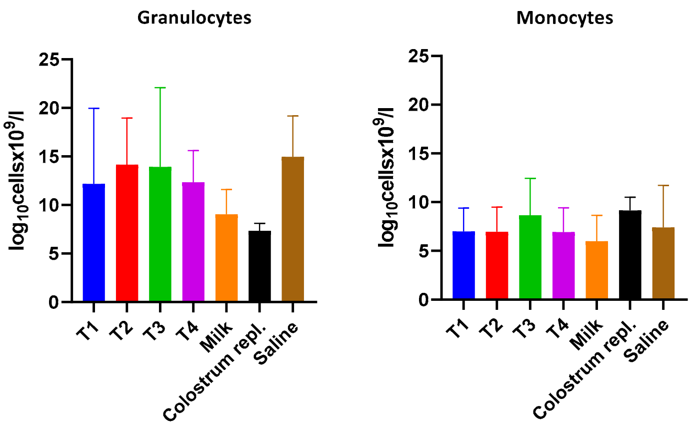

Figure S2. Early administration of colostrum fractions collected at different time points (1-4 hours) after partition does not induce the significant changes in granulocyte and monocyte counts in Wistar rats. The 6 weeks old Wistar rats received colostrum, artificial colostrum replacer, milk and saline daily for 7 days. The weight changes were monitored. The results are shown as mean ±SEM from five experimental animals.

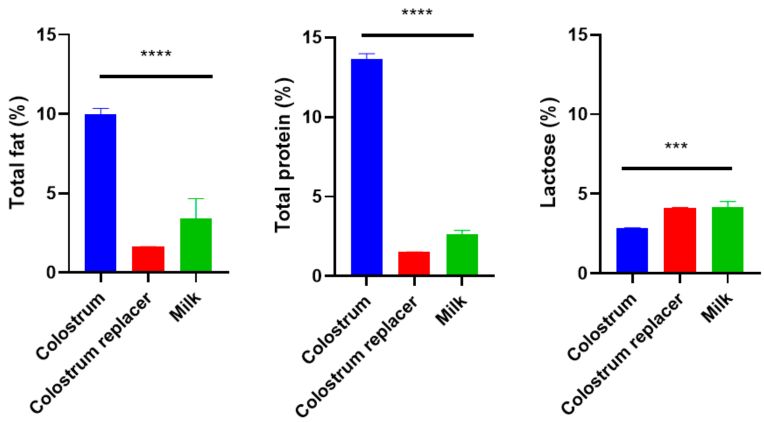

Figure S3. The nutritional profiles of bovine colostrum, colostrum replacer and milk. The results are shown as mean ±SEM from 3 samples.
